# Phenolic Composition, Mineral Content, and Beneficial Bioactivities of Leaf Extracts from Black Currant (*Ribes nigrum* L.), Raspberry (*Rubus idaeus*), and Aronia (*Aronia melanocarpa*)

**DOI:** 10.3390/nu12020463

**Published:** 2020-02-12

**Authors:** Monika Staszowska-Karkut, Małgorzata Materska

**Affiliations:** Department of Chemistry, Faculty of Food Science and Biotechnology, University of Life Sciences in Lublin, Akademicka 15 Street, 20–950 Lublin, Poland; monika.staszowska-karkut@up.lublin.pl

**Keywords:** black currant, raspberry, aronia leaves, phenolic compounds bioactivity

## Abstract

Currently, the incidence of lifestyle diseases is increasing due to inappropriate nutrition and environmental pollution. To prevent these diseases, various groups of bioactive compounds are needed with a broad spectrum of action and without adverse side effects. Polyphenolic compounds are the most widely studied group of this type of compounds. They occur widely in plants, but their content depends on many factors, including the type of plant, climatic conditions, and the date of harvest. The spectrum of bioactivity of phenolic compounds is determined by their chemical structure, concentration, and interaction with other compounds. Traditional recipes have been studied to search for active plant ingredients. Leaves of shrubs and fruit trees were used in folk medicine as a panacea for many diseases and have been forgotten with time, but their benefits are now being rediscovered. In recent years, much new information about biological activity of phenolic compounds from berry bushes (black currant, raspberry, and aronia) was published. This was reviewed and discussed in this article. The mineral content of their leaves was also summarized because minerals constitute a significant component of plant infusions. It has been shown that high antioxidant and biological activity of leaf extracts results from the presence of active phenolic compounds, which occur in definitely higher amounts than in fruits. Therefore, the leaves of berry bushes seem to be a promising source of substances that can be used as replacements for synthetic agents in the treatment and prevention of lifestyle diseases.

## 1. Introduction

In recent years, plant extracts and natural bioactive compounds have increasingly replaced synthetic antioxidants as components in functional food products and in pharmaceutical and cosmetic preparations [1,2]. This is due to the market demand for plant preparations, which may replace synthetic components whose use must be limited. Therefore, research on bioactivity and chemical characterization of various plant materials is of considerable scientific and practical importance. On the one hand, the search for active compounds in plants is quite simple because active substances occur in every anatomical part of the plant. On the other hand, because of a huge variety of plant species growing on the Earth, the search for bioactive compounds with directional effects is tedious and not always successful. The solution to this issue may be the use of traditional and folk medicine recipes developed using plants. For decades, people have used medicinal plants without knowledge of their chemical composition. Currently, on the basis of these traditional practices, scientists have extracted active ingredients from plants and have investigated their potential use in various industries [3].

Many degenerative diseases such cancer, atherosclerosis, and diabetes mellitus result from the harmful effects of free radicals on cellular systems [4]. Plant extracts contain bioactive compounds that act as antioxidants and can neutralize the activity of free radicals. Hence, antioxidants are considered as preventive compounds because they can repair the damage caused by free radicals [4]. A large group of biologically active compounds are phenolic compounds. These compounds are secondary plant metabolites that contain benzene rings with one or more hydroxyl substituents, ranging from simple phenolic molecules to highly polymerized structures [5,6]. Phenolic compounds have the strongest antioxidant and antiradical properties among all secondary metabolites; they react with a wide range of free radicals, including hydroxyls, superoxide anions, and other organic and nonorganic radicals. Polyphenols may also enhance the action of other antioxidants, including fat-soluble vitamins and low-molecular-weight water-soluble substances [1,6].

Fruits and vegetables are good sources of broad-spectrum bioactive compounds. Black currants, raspberries, and chokeberries are temperate plants that perfectly bear fruit in Polish climatic conditions. Additionally, according to FAOSTAT data [7], Poland ranks second in the world, after Russia, in the production of currants, mainly black currants. The production of these fruits is of great economic importance for Poland, because their export in fresh and frozen form or their products (juices, jams) comes second after the export of apples. Currants are well-known fruits and are commonly used in the food and pharmaceutical industry. Knowledge about the healing properties of these fruits has been transferred across many generations. Black currant (*Ribes nigrum* L.) is recognized as a good source of polyphenols, especially anthocyanins, phenolic acid derivatives, flavonols, and proanthocyanidins (PACs) [8]. Red raspberry (*Rubus idaeus* L.) is a popular soft fruit grown in Eastern Europe. Raspberry syrup is a traditional antipyretic and diaphoretic drug [9]. Berries of *Aronia melanocarpa*, also called aronia or black chokeberry, are known for their richness in procyanidins and anthocyanins, which are used as natural food colorants [2].

In recent years, several reports have been published on the use of anatomical parts of these plants other than the fruits as a source of active phytochemicals [3,10,11,12,13]. Studies on the potential of leaves, which are plant waste, demonstrate their usage in accordance with “green chemistry” principles. This article presents the current state of knowledge on individual phenolic and mineral compounds found in the leaves of black currant, raspberry, and aronia as a valuable source of bioactive substances with antimicrobial, antioxidant, and health-promoting properties.

## 2. Phenolic Compounds

**Figure 1:**
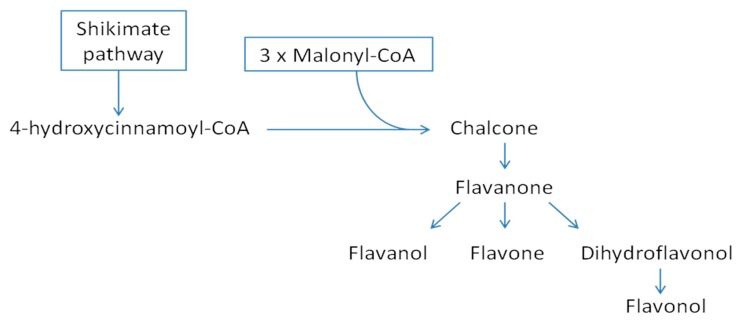
Scheme of the flavonoid biosynthetic pathway [16].

Phenolic compounds are generally divided into two groups: Phenolic acids and flavonoids, among which there are a few subclasses [14]. Most of the phenolics occur in nature as bound forms combined with sugars, organic acids, and esters, although some phenolics occur as aglycones. Phenolic acids are found in plant tissues primarily as hydroxyl derivatives of benzoic and cinnamic acids. These compounds produce the bitter and sour taste of plants, and they have astringent properties [15]. They are substrates in biosynthesis reactions (e.g., caffeic acid is a precursor of lignin) [14]. In addition to phenolic acids, flavonoids constitute the largest group of polyphenolic compounds. According to their chemical structure, they are divided into several sub-classes: Flavanones, flavanols, flavones, isoflavones, flavonols, and anthocyanins [16]. The basic skeleton of flavonoids is composed of 15 carbon atoms that form a C6-C3-C6 moiety, which can be interpreted as benzene ring system A, biogenetically derived from three active acetate molecules, and a C6-C3 system (B + C ring) formed from the shikimic acid pathway (Figure 1). Most flavonoids contain a heterocyclic system between aromatic rings A and B [16]. Individual flavonoids differ in the number, type, and location of the substituents in the molecule, which determine their chemical and physical properties and affect the individual metabolism and biological activity of each compound. Flavonoids are present in plants in two forms: Free aglycones and bound forms as *O*- and *C*-glycosides [14,16]. They have a high ability to absorb UV radiation, which indicates that they offer protection against its harmful effects. The protective function of flavonoids also involves the quenching of reactive oxygen species (ROS) produced in plants in increased amounts under stress conditions. Flavonoids regulate the activity of many enzymes, including those involved in the formation of ROS, e.g., peroxidase, lipoxygenases, and xanthine oxidase [14,16].

The concentration of phenolic compounds in plants depends on many factors such as cultivar, cultivation method, place of occurrence, weather conditions, and harvest time; additionally, the methods of extraction and analysis affect the final results. Therefore, comparison of the results of different experimental studies is difficult. However, on the basis of available literature data, it can be stated that the richest in total phenolics were aronia leaves (50–251 mg galic acid/g DM) [17,18] and the second were raspberry leaves (84–144 mg GA/g of DM) [19]. The lowest levels of phenolic compounds were noted in black currant leaves (40–78 mg GA/g DM) [20,21]. Concentration of phenolic compounds in black currant, raspberry, and aronia leaves is comparable to their levels in leaves of other plants, but generally, leaves of wild shrubs contain more of phenolics than leaves of cultivated shrubs [10,11,19,22]. This tendency was proven in Milenkovic-Andjelkovic et al. [23]. They analyzed the total phenolic content in leaves of four domestic species of fruit bushes (red currant, black currant, raspberry, and blackberry) and five wild species (European cornel, dog rose, hawthorn, blackthorn, and blackberry) and noted almost 20% higher level of those compounds in leaves of wild species. Teleszko and Wojdyło [10] compared the content of phenolic compounds in leaves of seven plant species and showed that the aronia leaves after the leaves of quince and cranberry were richest in these compounds. These results support the thesis that wild plants contain a higher concentration of secondary metabolites than domestic species. In turn, Skupień et al. [22] showed the highest content of total phenolics in raspberry leaves (525 g/100 g) in comparison with strawberry (373/100 g) and blueberry (111.5/100 g) leaves. On this basis, it can be concluded that among the three species of plants discussed, the best sources of phenolic compounds are aronia and raspberry leaves.

Usually, the content of phenolic compounds is higher in the leaves than in the fruits of the plant. Tabart et al. [24] found this pattern for leaves of black currant and noted that leaves had a different profile of bioactive compounds than berries. These results were also confirmed by Teleszko and Wojdyło [10] in raspberry and aronia plants. Cvetanovic et al. [25] compared the biological potential and chemical composition of extracts from the stems, leaves, and berries of *A. melanocarpa* and showed that the highest concentration of total phenolic compounds (131.53 mg of chlorogenic acid equivalent/g) and flavonoid (88.64 mg of rutin equivalent/g) was present in leaves, followed by stems and berries. Paunovic et al. [8] showed that soil management systems had positive effects on the synthesis and accumulation of flavonols and flavan-3-ols in both berries and leaves of black currant and a positive influence on anthocyanin accumulation in leaves but not in berries. Nour et al. [20] examined changes in the content of phenolic compounds in black currant leaves collected on several dates of the growing season and noted the highest total phenolic content (40 mg of gallic acid equivalent/g) and antioxidant capacity in leaves of six black currant cultivars collected in mid-June. Furthermore, Vagiri et al. [21] documented the highest level of total phenols in black currants at the end of August (87 mg GA/g DW), but the content of several phenolic compounds was highest in June. Similarly, for aronia, leaves harvested in September showed higher content of phenolic compounds and antiradical activity than those noted in leaves harvested in July [18]. Additionally, Cvetkovic et al. [2] investigated the possibility of using aronia leaves in the senescence vegetative stage, when the leaves are considered as an agriculture waste, as a potential source of bioactive compounds. They noted that in aronia leaves collected in November, the total content of phenolic compounds was 15 mg of gallic acid equivalent/g. These results prove that the synthesis of phenolic compounds occurs throughout the entire growing season, and the accumulation time varies between plant species. Fruits of black currant and some of raspberry varieties are usually harvested in the first half of July. This date may be also considered as a convenient time for leaf collection. However, in the case of industrial-scale harvesting (total leaf harvesting), it should be taken into account that this treatment will have a significant effect on the plant and its yielding in the next year. Therefore, leaf collection may be recommended for shrubs to be removed.

In recent decades, the most common method used for the separation, quantitation, and identification of natural compounds is a combination of HPLC techniques with spectrophotometric (DAD) or spectroscopic (MS) detection [10,11,26,27]. The results of quantitative analysis of phenolic compounds vary among studies. In addition to the abovementioned varietal and climatic factors, methods of sample preparation and analysis also influenced the final results. The results of the quantitative analysis of individual phenolic compounds are converted into the mass of dry leaves or to the weight of the dry extract (Table 1). This conversion leads to difficulty in interpreting data from the literature. Nevertheless, the main phenolic acids quantified in black currant leaves were chlorogenic, neochlorogenic, cryptochlorogenic, and *p*-coumaric acids, with the most abundant being *p*-coumaric acid [8,10,20,21,26]. *Rubus* leaves were rich in ellagic acid and *p*-coumaric acid [12,19,23,28,29], while in aronia leaves, the highest levels of sinapic acid were identified [25]. Other authors stated that the main phenolic acids occurring in aronia leaves were chlorogenic, neochlorogenic, 3,4- dihydroxyphenylacetic, and protocatechuic acids [18,30] (Table 1). There are few data in the literature comparing phenolic acids profile in the leaves of various species of fruit bushes. On the base of available data, the content of individual phenolic acids in the leaves of blackcurrant, raspberry, and aronia with their content in the leaves of other plants, it can be stated that each species has a specific profile of these compounds. Skupień et al. [22] showed that ellagic, p-coumaric, and caffeic acid dominated in raspberry leaves in comparison to strawberry and blueberry leaves, while the highest level of gallic acid was noted in strawberry leaves [22]. Oszmianski et al. [26], based on comparative analysis, showed that black currant leaves were good source of chlorogenic acid and among the leaves of raspberry, bilberry, and strawberry, only bilberry contained more of this compound than black currant. Additionally, Milenkovic-Andjelkovic et al. [23] showed that chlorogenic acid dominated in leaves of domestic species and the highest level of this compound was noted in red currant leaves [23].

In addition to flavonoids, quercetin-3-*O*-derivatives were most predominant in black currant and raspberry leaves, while quercetin-3-*O*-glucosyl-6′-acetate and quercetin-3-*O*-glucuronide occurred mainly in black currant and raspberry leaves, respectively [26,29]. Other flavonoids present in black currant leaves were kaempferol and myricetin as well as their derivatives [24]. The concentration of flavonoids in aronia leaves was lower than that in black currant and raspberry leaves (Table 1). Similar to that noted in black currant and raspberry leaves, the most abundant compound in this group of compounds was quercetin and its derivatives [18,23,25]. As in the case of phenolic acids, the concentration of these compounds in the leaves of wild species was usually higher than in the case of domestic species [23,26]. The concentration of quercetin derivatives in black currant and raspberry leaves was on a similar level to in fruits of red pepper, however there are differences in the types of sugar substituents to quercetin [31].

## 3. Macro- and Microelements

Minerals and trace elements play an important role in the activation of enzymes involved in cell metabolism and antioxidant systems [32]. High intake of K, Mg, and Ca has been linked to reduced risk of stroke, hypertension, and osteoporosis [33]. Iron and other micro minerals are an essential part of many compounds in the oxygen transport and storage system and function as cofactors for enzymes [33,34,35]. Micronutrient Mo is a structural component of xanthine oxidase and xanthine dehydrogenase, which are crucial for urea synthesis [2].

The leaves of berry plants contain minerals such as potassium (K), calcium (Ca), magnesium (Mg), phosphorus (P), sodium (Na), and iron (Fe) and the trace elements such as copper (Cu), zinc (Zn), manganese (Mn), and boron (B). The daily values of the amounts of minerals recommended per day by Food and Drug Administration (FDA) are: 3.5 (K), 1(Ca), 0.4 (Mg), 1(P), 2.4(Na), 0.018(Fe) g/day [2,36]. Leaf extracts consumed directly as infusions or used as food additives can increase the amount of macro and micronutrients in foods. Among the discussed plants, the highest content of Ca was found in black currant leaves, which also contained the most of Mg, P, and Fe. Raspberry leaves contained the most of K, B, and Na and chokeberry leaves the most of Mn. The contents of macro- and micronutrients in the leaves of black currant, raspberry, and aronia are summarized in Table 2. Mineral content in plants largely depends on the conditions of growth, including cultivation techniques, abiotic or biotic stress, and nutritional status [37]. Nour et al. [20] investigated the effect of the harvest time on the accumulation of minerals in black currant leaves and found highest concentrations of Ca, followed by K and Mg ions. The highest concentration of these ions was found in the leaves collected in mid-June. In addition, the content of microelements was the highest in black currant leaves collected in June, with Fe and Mn showing the highest levels [20]. Similar results were obtained by Niskanen et al. [37], who investigated the effect of mineral soil fertilization on the content of macroelements in black currant leaves. Karaklajić-Stajić et al. [38] studied micronutrients in raspberry leaves, and they found that Fe and Mn were present in the highest concentration among four investigated microelements. Biel and Jaroszewska [39] also stated that among 10 analyzed microelements, Fe and Mn occurred in the highest concentration in raspberry leaves. Further, the same authors noted the highest level of K followed by that of Ca in raspberry leaves. Aronia leaves are also a good source of macro- and micronutrients; this feature is particularly important when infusions from dried leaves are prepared because the minerals are easily extracted into the solution. Cvetkovic et al. [2] found that the main macronutrient present in the aronia leaf extract was P, followed by Mg, K, Na, and Ca. Among the micronutrients, the main micronutrient was Fe, followed by Zn, Mn, and Cu. Other authors [35] who analyzed the chemical composition of aronia leaves showed lower content of macronutrients but higher content of Fe and Zn than those reported by Cvetkowic et al. [2]. The differences in the element concentrations in leaves were explained by the differences in the composition of soil and climatic conditions in which the plants were grown. The leaves usually contain more minerals compared to fruit. This was confirmed by the research of Pavlovic et al. [34], where the mineral composition of aronia fruit and leaves and their products was compared, and higher concentrations in fruit than in leaves only for K were found.

## 4. Antioxidant Potential

Several studies have suggested that phenolic compound content, antioxidant activity, and anticancer activity are closely related to each other [4,40]. Thus, investigation of the antioxidant activity of natural products is the first step in the search for bioactive substances in plant extracts. Many methods are available for determining the antioxidant activity, but the most commonly used one is the DPPH radical assay. Extracts from black currant, raspberry, and aronia leaves have been thoroughly tested for their antioxidant activity [10,11,17,41]. For extracts from black currant leaves, the younger leaves showed a higher antioxidant activity than those collected at later stages of growth [17]. Furthermore, Nour et al. [20] stated that extracts from leaves harvested in mid-June showed the highest antioxidant activity, which was correlated with the total phenolic content. By comparing three types of extraction methods, these authors showed that the extraction solvents significantly influence the total phenolic content and the antioxidant capacity. The best solvent for the extraction of bioactive compounds from black currant leaves was 40% ethanol [20]. Tian et al. [11] found that acidified ethanol-water extracts from fruits and leaves of berry plants showed variable free radical scavenging activity, according to the DPPH radical assay, and oxygen radical absorbance capacity (ORAC); they also showed that leaf extracts exhibited higher antioxidant activity than extracts from berries. These authors noted higher antioxidant activity of the extracts prepared from raspberry leaves than that of extracts from aronia leaves [11].

Different antioxidant activity of the extracts obtained from various plant parts was shown by Cvetanovic et al. [25]. These authors demonstrated that in the DPPH assay, extracts from aronia leaves showed the highest antiradical potential that extracts from aronia berries and stems. Similarly, other studies also found high antiradical activity of aronia leaf extracts in the DPPH radical assay [18].

## 5. Effect on Enzyme Activity

Parallel to classical analyses, including studies of antioxidant activity of black currant leaf extracts, Tabart et al. [42] conducted a series of in vitro tests on cellular models. In studies on anti-inflammatory capacity, these authors evaluated the effect of the extract on the activity of myeloperoxidase (MPO), which was released from stimulated neutrophils to mimic acute inflammation resulting in ROS production. They noted that compounds contained in the extract could scavenge ROS produced by neutrophils or inhibit the activity of MPO [42]. In other investigations, the antithrombotic function of human umbilical vein endothelial cells was tested in the presence of black currant leaf extracts [43]. It was observed that the extracts enhanced endothelial nitric oxide synthase (eNOS) activation and thus possibly improved endothelial cell viability at low physiological concentrations without affecting the antiplatelet action of endothelium [43].

Extracts from aronia were tested for inhibitory activity of enzymes linked to Alzheimer’s disease. It was observed that aronia leaves were active inhibitors of acetylcholinesterase but not of butyrylcholinesterase. In these investigations, the inhibitory activity of extracts from aronia leaves was comparable to that of extracts from aronia fruits [25]. Elastase was another enzyme to be tested with extracts from aronia fruits and leaves. Elastase is the main enzyme that causes the breakdown of elastin, an important protein in the extracellular matrix of cells. Therefore, research on elastase inhibitors may provide a new material for developing novel cosmetics. Although extracts from aronia leaves showed inhibitory effects on this enzyme, the effects were lower than those of extracts from berries and stems [25]. Table 3 shows a summary of the influence of berry leaf extracts on enzyme activity.

## 6. Cytotoxic Activity

Between the three types of leaf extracts discussed here, the aronia leaf extracts were the most analyzed ones for their cytotoxic activity on many cancer cell lines (Table 3). Cvetanovic et al. [25] investigated three types of malignant cell lines (A-549, LS-174T, and HeLa) and normal pulmonary fibroblasts (MRC5) and found that aronia leaf extracts showed the highest cytotoxic activity as compared to extracts obtained from other parts of this plant. The authors suggested that the large amounts of phenols and flavonoids in aronia leaf extracts are responsible for better cytotoxicity effects. Skupień et al. [30] evaluated the antileukaemic activity of hydrolysed aronia leaf extract on the HL60 cell line and its multidrug-resistant sublines HL60/VINC and HL60/DOX. The authors found that the aronia extract inhibited the growth of the sensitive leukaemic cell line. Thi and Hwang [17] studied the anticancer effect of aronia leaf extracts on SK-Hep1 human hepatoma cells and demonstrated that the extracts inhibited cell growth and metastasis of cancer cells in dose-dependent manner.

Skupień et al. [22] demonstrated that extracts from raspberry leaves exhibited high cytotoxic activity against the sensitive leukaemia HL60 line (IC_50_ = 0.380 g/L) and that this cell line showed a very low value of the resistance factor for this extract (RF = 0.34). Additionally, they estimated the contribution of individual phenolic compounds to the total antileukemic activity of the analyzed leaf extracts. They confirmed relatively good correlations between the contents of ellagic acid, quercetin, and gallic acid and the ability of extracts to inhibit the growth of the sensitive HL60 cell line and its sublines [22]. The bioactive potential of red raspberry leaf extract was also investigated by Durgo et al. [44]. These authors analyzed the cytotoxic effect of the leaf extract on human laryngeal carcinoma (HEp2) and colon adenocarcinoma (SW480) cell lines. They noted cytotoxic effect on both HEp2 and SW480 cell lines, but SW480 cells were more susceptible to raspberry leaf extracts than HEp2 cells [44].

## 7. Antimicrobial and Antifungal Activities

The antimicrobial activity of black currant fruit and leaf extracts was estimated against three gram-positive and three gram-negative bacterial cultures and nine types of fungi by using the method reported by Paunovic et al. [8]. By comparing the minimum inhibitory concentration (MIC) values of extracts from leaves and berries of seven types of black currant, the authors noted antimicrobial activity of the extract from leaves against *Proteus vulgaris*, *Candida albicans,* and *Aspergillus niger*, but the activity was lower than that for extracts from berries [8]. Raudsepp et al. [45] also investigated the antibacterial activity of different plant extracts against four gram-negative and five gram-positive bacterial species; they noted that gram-negative bacteria were less susceptible to plant infusions than gram-positive bacteria. They noted the strongest antibacterial activity of extracts from black currant leaves against the gram-negative bacterial species *Campylobacter jejuni* and *Yersinia ruckeri* and the gram-positive bacterial species *Bacillus cereus* and *Listeria monocytogenes* [45]. Milenkovic–Andjelkovic et al. [23] investigated the antimicrobial activity against 13 microbial species and found that black currant leaf extracts showed higher antimicrobial activity than raspberry leaf extracts. They also noted that the leaf extracts had greater antimicrobial effects on gram-positive strains than on gram-negative strains. The inhibitory effects of the compounds contained in raspberry leaves on *L. monocytogenes* was confirmed by Tian et al. [11].

Cvetanovic et al. [25] tested the antibacterial activity of extracts from aronia leaves against two gram-positive and four gram-negative bacterial strains and determined the antifungal activity of the extract against two fungal species. The authors compared the antimicrobial activity of aronia extracts with that of amracin (a tetracycline antibiotic). Compared to amracin, the leaf extracts showed four times stronger antibacterial activity against *P. vulgaris* and 15 times stronger activity against *Proteus mirabilis*. Moreover, the antifungal activity of aronia leaf extracts was comparable to that of nystatin (an antifungal medicine) [25].

Similar to antioxidant activity, the antibacterial activity of plant extracts results from the presence of phenolic compounds. Tian et al. [11] showed positive correlations between the sum of phenolic compounds and antibacterial activities against *Staphylococcus aureus* and *Bacillis cereus*. They noted that ellagitannins and isorhamnetin di- and tri-glycosides were the main inhibitors and stated that the compositional profiles play a major role in the anti-bacterial activities of the plant extracts [11]. Some authors demonstrated that the number of hydroxyl groups in the molecules might affect the antimicrobial activity of phenolic compounds and glycosilation of flavonols may reduce the efficacy on growth inhibition [46,47]. Alves et al. [48] reported that the antibacterial capacity of phenolic acids mainly dependent on the presence of carboxyl group and the substitution pattern in benzene ring [48]. These examples indicate the differences in bioactivity among groups of phenolic compounds and justifies screening for plants and their extracts for targeted applications as food preservatives and as a medical application.

## 8. Health-Promoting Properties and Application in Medicine

In traditional folk medicine, infusions from the leaves of berry bushes were used for many therapeutic purposes. Black currant leaf infusions were used to fasten the process of excretion of toxins from the body and to regulate kidney function [49]. These extracts were used as diaphoretic and diuretic agents as well as for the treatment of inflammatory disorders such as rheumatic disease [20,24]. Leaf tea of *R. idaeus* L. (raspberry) has been used for centuries in folk medicine as a panacea for diarrhea and colic. The infusion of raspberry leaves was used in compresses and poultices for skin diseases [3,13]. Aronia was used in Siberia and by American Indians as an elixir of youth, which sharpened the mind and helped to heal bone injuries. Frequently, aronia was given to young women, especially during pregnancy, because it promoted strength development [13]. In the past, aronia leaves were used in traditional medicine as anti-inflammatory, antiviral, antibacterial, and anti-proliferative agents [25].

Several studies indicate that the health-promoting properties of leaf extracts result from the presence of a number of active compounds, mainly phenolics, which show a wide spectrum of activity. Flavonoids have antiatherosclerotic activity and inhibit atherosclerosis formation in many stages of pathogenesis [50]. Studies have shown that flavonoids lower cholesterol levels in blood [51]. It has also been observed that the majority of phenolic compounds have an antidiabetic effect, although the mechanisms of action differ. Further, naringin and hesperidin were found to play important roles in preventing the progression of hyperglycaemia, partly by increasing hepatic glycolysis and glycogen concentration and/or by lowering hepatic gluconeogenesis [52]. Rutoside has also been shown to have potent hypoglycaemic and hypolipidaemic effects by increasing the peripheral use of glucose by skeletal muscles and stimulating beta cells [53]. In addition, the antihyperglycaemic effect of luteolin 7-O-glucoside, hyperoside, and isoorientin was confirmed [54,55]. Brahmachari [56] showed that isoquercetin inhibits nonenzymatic N-glycosylation of proteins, and its activity was comparable to that of aminoguanidine.

Studies in recent decades clearly confirmed that extracts from black currant leaves have a beneficial effect on health, mainly through anti-inflammatory and antioxidant effects [24,49,57]. The influence of water-alcoholic black currant leaf extracts (*R. nigrum*) was investigated on carrageenan-induced rat paw oedema. Pharmacological activity was compared with indomethacin and niflumic acid by using orally acute and chronic treatment (21 or 28 days) [57]. The inhibitory effects of PACs isolated from *R. nigrum* leaves on carrageenan-induced inflammation in rats were also investigated. PACs have been shown to interfere with the accumulation of circulating leucocytes associated with the reduction of proinflammatory factors such as TNF-α, IL-1β, and CINC-1 [57]. Black currant extract showed significant anti-inflammatory activity comparable to that observed for reference substances, but without their ulcerative potential, even at high doses during long-term treatment [49].

Extracts of raspberry leaves inhibit bacterial growth, lower blood glucose, and lipid levels, and show anticoagulant activity [58,59]. Extracts of dried raspberry leaves prepared with different solvents (n-hexane, ethyl acetate, chloroform, and methanol) were tested in vitro for producing a relaxant effect on stimulated guinea pig ileum. The highest activity was found for methanol extracts, which demonstrated the polar nature of the bioactive compounds [60]. Investigations conducted by Han et al. [58] on antithrombotic activity of compounds derived from raspberry leaf extracts confirmed the anticoagulant activity of the extracts in both in vitro and in vivo tests. Further studies revealed that the active ingredients of raspberry leaf extracts were kaempferol, quercetin, and tiliroside [58].

Aronia leaf extracts have been used as a source of active ingredients for biopharmaceutical engineering [61]. A previous study investigated the reparative properties of aronia leaf extract for skin damage in female New Zealand rabbits. The leaf extracts were applied to the damaged tissue for seven days. In vivo tests showed complete epithelialization of the damaged sites and reduction in erythema and oedema [61]. The antioxidant activity of aronia leaf extract on the brain was also examined [62]. Intraperitoneal injection of the extract at a dose of 0.2 g/kg prevented symptoms of oxidative stress caused by immobilization in the brains of rats. Oral administration experiments revealed that the extract reduced the intensity of peroxidation of lipids and proteins induced by ascorbate and H_2_O_2_ in brain homogenates. The highest effect of the extract on ascorbate-induced lipid peroxidation was found in the post-mitochondrial fraction of the rat brain homogenate [62]. Intraperitoneal or oral administration of aronia leaf extract significantly reduced blood glucose levels in healthy rats and in animals with diabetes mellitus induced by streptozotocin. Extracts from aronia leaves were shown to stimulate glucose use by cells of PC12 and L929 cell lines [63,64].

The biological effects of polyphenolic compounds are limited by their low absorption from the digestive tract [65,66]. It has been shown that chemical structure determined the rate and extent of their intestinal absorption. For example, the ferulic and sinapic acids are absorbed as free and soluble forms, while bigger molecules must be firstly hydrolyzed by enteric enzymes or bacterial enzymes of colon microflora [65]. The main phenolics detected in leaves extracts are small compounds, such as gallic acid in black currant, ellagic acid in raspberry, and chlorogenic acid in aronia leaves (Table 1). On this base, it can be said that phenolic compounds from leaves of fruit bushes could show good bioavailability, but the further investigations are needed to prove this statement.

## 9. Summary

The substances contained in the leaves of berry bushes, particularly polyphenolic compounds, have properties that benefit health. These substances have potential use in the prevention of lifestyle diseases. Biological effects of extracts from the leaves of black currant, raspberry, and aronia are associated with their high antioxidant activity, which was confirmed in many in vitro and in vivo analyses. Therefore, the leaves of berry bushes are a promising source of bioactive substances that can be used as replacements for synthetic agents in the treatment and prevention of lifestyle diseases. They may also be used as valuable food additives, thus increasing the functional qualities of food.

## Figures and Tables

**Table 1:** Distribution of phenolic compounds in the leaves of black currant, raspberry, and aronia (mg/100 g dry mass (DM)).

Compound	Black Currant		Raspberry		Aronia	
	Leaves	Extract	Leaves	Extract	Leaves	Extract
Caffeic acid	9 [20]		0.3–77 [13,19,22,28]	27 [23]	52 [30]	
Chlorogenic acid	1–10 [20,21]	21 [23]	2.9–23 [13,28,29]	39 [23]	64–706 [18,30]	3 [25]
Neochlorogenic acid	14 [21]		13–17 [19]		41–483 [18,30]	
Ferulic acid	2 [20]		17.6–19 [19]			5 [25]
Gallic acid	20 [20]	18 [23]	2.3–31 [19,22]	27 [23]		
p-Coumaric acid	29 [20]		0.9–67 [19,22,28]		4 [30]	9 [25]
Salicylic acid	24 [20]		41 [28]			
Sinapic acid	1 [20]					55 [25]
Rosmarinic acid			1–3 [13]		23–155 [18]	16 [25]
Ellagic acid		415 [23]	19–281 [19,22,28,29]	438 [23]		
Apigenin						21 [25]
Luteolin			49 [28]			33 [25]
Quercetin	5–136 [20,24]	352 [23]	2–62 [19,22,28]	301 [23]	29–316 [18,30]	11 [25]
Quercetin 3-*O*-rutinoside	16–210 [20,21,24]	584 [23]	5–59 [13,19,28,29]	478 [23]	62–103 [18]	69 [25]
Quercetin 3-*O*-galactoside	7 [21]		3–72 [13]			
Quercetin 3-*O*-glucoside	5–132 [21,24]	714 [23]	83 [28]	811 [23]		
Quercetin 3-*O*-malonylglucoside	301 [21]					
Kaempferol	1 [24]		0.5–37 [19,22]			7 [25]
Kaempferol 3-*O*-rutinoside	2 [21]		3 [29]			
Kaempferol 3-*O*-glucoside	2–75 [21,24]	410 [23]	27–126 [13]	278 [23]		
Myricetin	8–83 [20,24]		23 [28]			
Myricetin 3-*O*-malonylglucoside	5 [21]					
Catechin		92 [23]	169–191 [19]	247 [23]		
Epicatechin		127 [23]	0.4–13 [19,29]	378 [23]		
Epigallocatechin	1 [21]	46 [23]	0.5–1.5 [19]			
Procyanidin B2		278 [23]				

**Table 2:** Macro- and microelement content in the leaves of black currant, raspberry, and aronia (mg/g DM).

Element	Black	Raspberry	Aronia
	Currant		
N	24 [37]	22 [39]	18.5 [39]
Ca	17–21 [20,37]	8 [39]	3.6–9.2 [2,35,39]
Mg	5–6 [20,37]	5.4 [39]	0.8–4.7 [2,35,39]
K	11–17 [20,37]	17 [39]	0.76–15.8 [2,35,39]
B	0.034–0.06 [20,37]	0.098 [38]	0.005 [35]
Fe	0.28–0.43 [20]	0.064–0.183 [38,39]	0.017–0.023 [2,35,39]
Mn	0.041–0.077 [20]	0.064 [39]	0.006–0.151 [2,35,39]
P	6 [37]	4 [39]	1–3.3 [2,35,39]
Na	0.013–0.063 [20]	4 [39]	0.018–0.35 [2,35,39]
Mo		0.004–0.021 [38,39]	0.0008–0.025 [35,39]
Cu	0.004–0.007 [20]	0.004–0.029 [38,39]	0.001–0.02 [2,35,39]
Zn		0.03 [39]	0.008–0.025 [2,35,39]
Al	0.1–0.19 [20]		0.011 [35]
Cr	0.003–0.004 [20]	0.001 [39]	0.0005–0.001 [35,39]
Ni		0.005 [39]	0.00014–0.004 [35,39]
Cd		0.003 [39]	0.00002–0.003 [35,39]
Pb		0.006 [39]	0.0001–0.009 [35,39]
Co		0.0004 [39]	0.0005 [39]

**Table 3:** Biological activity of extracts from black currant, raspberry, and aronia leaves.

Biological Activity	Black Currant	Raspberry	Aronia
Enzymes inhibition Enzymes enhance	Myeloperoxidase (MPO) [42] Nitric oxide synthase (eNOS) [43]		Acetylocholinoesterase [25] Elastase [25]
Cytotoxic effects		HCT-116 [41] HEp2 [44] HL60 [22] SW 480 [44]	A-549 [25] HeLa [25] HL60 [18,30] L1210 [18] LS-174T [25] SK-Hep1 [17]
Antibacterial activity	*Aspergillus niger* [8] *Bacillus cereu* [45] *Campylobacter jejuni* [45] *Candida albicans* [8] *Listeria monocytogenes* [23,45] *Proteus vulgaris* [8] *Sarcina lutea* [23] *Staphyloccus aureus* [23] *Yersinia ruckeri* [45]	*Listeria monocytogenes* [11,23] *Sarcina lutea* [23] *Staphyloccus aureus* [11,23] *Salmonella enterica* [11]	*Listeria monocytogenes* [11] *Proteus mirabilis* [25] *Proteus vulgaris* [25] *Staphyloccus aureus* [11] *Salmonella enterica* [11]
